# Chronic Diseases Knowledge and Related Factors among the Elderly in Jinan, China

**DOI:** 10.1371/journal.pone.0068599

**Published:** 2013-06-24

**Authors:** Yapei Song, Wei Ma, Xiangren Yi, Shumei Wang, Xiaojie Sun, Jiming Tian, Shukang Wang, Chunmei Zheng, Bingyin Zhang, Zhijian Xu, Gifty Marley

**Affiliations:** 1 School of Public Health, Shandong University, Jinan, China; 2 The College of Physical Education, Shandong University, Jinan, China; 3 Center for Health Management and Policy, Shandong University, Jinan, China; University Heart Center, Germany

## Abstract

**Background:**

It has been reported that the prevalence of chronic diseases is high among old people and they have poor chronic diseases knowledge. This study was therefore designed to evaluate the awareness rate of chronic diseases knowledge among people aged over 60 years, to explore its related factors and to provide evidence for future health education.

**Methods:**

A cross-sectional study was conducted from April to August in 2011. People aged 60 years and above from 3 communities in Jinan were selected by cluster sampling. Nine hundred and twenty five participants were interviewed face-to-face using a structured questionnaire.

**Results:**

The awareness rates of chronic diseases knowledge varied from 29.5% to 90.2%. Four healthy lifestyles including quitting smoking and less drinking, keeping broad-minded, maintaining balanced diet and moderate physical activity were best known (from 86.3% to 90.2%). The least known knowledge were 2 complications of hypertension: nephropathy (29.5%) and retinopathy (37.2%). Participants with the following characteristics or behaviors were more likely to have higher chronic diseases knowledge: younger age, female, Han Chinese, higher level of education, having health insurance, participating in societies, having family history of chronic diseases, frequently gathering with friends/relatives, usually going to provincial hospitals/hospitals affiliated with medical universities, usually going to municipal hospitals and usually going to community health center/station.

**Conclusions:**

Old people in Jinan had incomplete chronic diseases knowledge and the overall awareness rate was not high. The older people’s chronic diseases knowledge should be improved and health education programs should target males, older people with lower educational level, having no health insurance, having no family history of chronic diseases, participating in no societies, and less frequently gathering with friends/relatives. Also, lower level medical facilities should improve their skills of health education.

## Introduction

A United Nations (UN) report showed that globally the population of older persons (aged 60 or over) is growing at a rate of 2.6% per year, considerably faster than the population as a whole (1.2%). In 2009, the number of older persons had surpassed 700 million. Older persons are projected to reach 2 billion by 2050, tripling the number in 2009 [1]. The pace of population ageing is faster in developing countries than in developed countries. The China National Committee on Ageing reported that China had become an ageing society since 1999, and predicted that the ageing population would surpass 400 million, accounting for 30% of the total population in 2050, which would result in a heavy burden on the national health care system because the consumption of medical and health resources of the old people is generally 3-5 times as much as that of the rest of the population [2].

With the epidemiological transitions, chronic diseases, which caused 63% of all deaths worldwide [3], have become serious public health problems in the world. Global concern about the growing global burden of non-communicable diseases prompted the United Nations to hold a high-level meeting on non-communicable diseases in New York in September 2011 [4]. The prevalence of chronic diseases is higher in older group [5–7]. It was reported that the prevalence of hypertension, diabetes mellitus (DM) were 49.1% and 6.8% respectively in Chinese old people aged over 60 in 2002 [8]. The incidence of all cancers of Chinese old people increased with age (from 708.64/10^5^ in 60-64 age group to 1635.25/10^5^ in 80-84 age group) in 2009 [9]. A study showed that the prevalence of chronic disease of the elderly in Shandong province was 81.8% and health care expenditure accounted for 28.8% in average daily consumption [10].

Chronic diseases exist in a long time, and usually can’t be cured [7]. Many of them are closely related to behavior and lifestyle, and can be prevented by changing unhealthy behaviors and lifestyle [11]. The World Health Organization (WHO) reported that six risk factors, including high blood pressure (BP), high blood glucose, overweight and obesity, physical inactivity, high cholesterol and low fruit and vegetable intakes, accounted for 19% of global deaths and have the greatest effect on cardiovascular diseases [12]. Experience from United States showed that the marked declines in death rates from coronary heart disease, stroke, all cardiovascular diseases, and all causes were related to lifestyle recommendation [13]. To promote behaviors, health knowledge must be improved firstly. Previous studies showed that patients’ better knowledge about diabetes/hypertension was associated with better medication adherence and better blood glucose/blood pressure control [14–16]. Cancer knowledge was a critical element in determining whether people would do cancer screening [17–19]. Therefore, improving health knowledge played an important role in chronic diseases prevention and control.

It was reported that 90% of community health service centers carried out health education in China [20]. However, some studies showed that the proportion of Chinese people with health literacy was very low and the group under 45 years old had higher health literacy than the group over 45 years old [21–23]. A study on a nationally representative sample of Chinese adults between 35 and 74 years old showed that only 23.7% diabetics were aware of their diabetes mellitus, 20.3% were taking prescribed medication or nonpharmacological interventions, and 8.3% had fasting plasma glucose <126 mg/dl [24]. Among hypertensive patients in Guangzhou, only 31.6% were aware of their high BP, 28.8% took medication, 1.2% took non-medication intervention and 12.6% achieved their BP under control (<140/90 mm Hg) [25]. Thus it’s urgent to evaluate the effect of health education in China, find its related factors, and develop effective health education strategy in order to control chronic diseases.

According to the third national death investigation [26], stroke and cancer were the two leading causes of death in China, and cancer was the first cause of death in cities. Globally, 51% of stroke and 45% of ischaemic heart disease were attributable to high systolic blood pressure, and raised blood glucose causes all diabetes deaths, 22% of ischaemic heart disease and 16% of stroke death [12]. Among chronic diseases, only hypertension and type 2 DM were provided standardized management by community health centers in China [27]. So knowledge of hypertension, DM and cancer were mainly considered in our study.

Despite the fact that some studies about chronic diseases of old people have been conducted in China, these studies were not comprehensive or profound enough [28–34]. Most of these studies concluded that the chronic disease knowledge of the elderly was not high and health education needs further reinforcement. However, few studies focused on factors influencing chronic diseases knowledge. Therefore, this study was designed to evaluate the awareness rate of chronic diseases knowledge among people aged over 60 years, to explore its related factors and to provide evidence for future health education.

## Methods

### Subjects

Subdistricts in the 3 communities were randomly selected. All eligible residents in selected subdistricts were invited to participate. Inclusion criteria for participants included: (1) age≥60 years, (2) able to answer the questionnaire, (3) living in the selected communities over 6 months in the past year. Individuals with cognitive impairment were excluded. A total of 1132 old people were invited. Among them, 925 were interviewed, and 207 were not involved in the survey because they refused to be interviewed or they were not available.

### Study Instrument

Mainly considering three chronic diseases (hypertension, DM and cancer) and the characteristics of old people, we designed a structured questionnaire which includes social demographic information, history and family history of chronic diseases, health-related behaviors, chronic diseases knowledge, the source of health knowledge and attitude to change unhealthy lifestyle. The researchers in our team estimated the content validity of the health knowledge, and thought that the questionnaire covered the basic knowledge of the three diseases. Fifty old people living in the selected subdistricts were investigated in pilot test.

Social demographic information included gender, ethnicity, age, marital status, educational level, employment status, health insurance status, living pattern, personal monthly income, main source of income and persons who provide care. Participants were also asked whether they were diagnosed with hypertension, DM, hyperlipemia, coronary diseases, stroke, cancer or other chronic diseases and whether their parents, brothers and sisters suffered from hypertension, coronary diseases, stroke, DM or other chronic diseases. Health-related behaviors included joining societies, participating in community activities, gathering with friends/relatives or neighbors, actions taken when being ill and types of medical facilities they usually went to.

Chronic diseases knowledge included healthy lifestyle, warning signs of cancer, threshold value of systolic and diastolic BP, risk factors of hypertension, control measures of hypertension, the threshold value of normal fasting plasma glucose and common knowledge of DM. Overall, there were 35 single choice questions about chronic diseases. A correct answer would get 1 point, and a false or missing answer would get 0 point. The total score was 35 points. A dichotomous variable was created for total knowledge by classifying participants with 21 or more points as higher knowledge (≥60% of the total points).

### Data Collection

A cross-sectional study was conducted from April to August 2011. Each participant was interviewed face-to-face by trained interviewers using the pilot tested questionnaires. Experienced investigators trained interviewers, monitored field survey and reviewed questionnaires.

### Data Analysis

The data was double input using Epidata 3.1 and analyzed using SPSS16.0 software. Proportion and ratio were used to describe participants’ characteristics and correct response to chronic diseases knowledge. According to tests of normality, total knowledge was non-normally distributed, thus nonparametric tests were used to compare total knowledge among subjects with different characteristics and behaviors. Mann–Whitney *U*-test and the Kruskal-Wallis test were used to compare 2 independent samples and K (K>2) independent samples separately. Binary logistic regression was used to analyze the factors related to higher knowledge. Independent variables included age, gender, ethnicity, marital status, educational level, employment status, health insurance status, living pattern, personal monthly income, main source of income, persons who provide care, family history of chronic diseases, suffering from chronic diseases, participating in societies, frequency of participating in community activities, frequency of gathering with friends/relatives, frequency of communicating with neighbors, actions when ill and medical facilities which they usually go to. Forward stepwise method was used to build the model, from which Odds ratios (OR) and corresponding 95% confidence intervals (CI) were derived. The P-values was two-tailed, with P<0.05 considered statistically significant.

### Ethics Statement

The study protocol was approved by the Institutional Review Board of Shandong University School of Public Health. Study procedure, voluntary nature of participation, participants’ right to withdraw and autonomy of the participants were explained and written informed consent was obtained from the participants before their interviews.

## Results

### Participants’ Characteristics

Of the 925 questionnaires, 803 were valid (the proportion of missing health knowledge was ≤ 25%). The effective rate was 86.8%. The majority of participants were female (65.4%), married (69.8%), Han Chinese (95.3%), retired (82.7%), living with others (86.0%), having education level of junior middle school and below (73.5%), having at least one health insurance (85.7%) and with their main source of income coming from themselves or spouse (87.6%). About half of participants were less than 70 years old, with personal monthly income between 1001 and 2000 Yuan and taken care by their spouse. More details were shown in Table 1.

**Table 1:** Demographic characteristics of the 803 elderly people in Jinan, China.

		n^*^	%
Gender			
	Male	278	34.6
	Female	525	65.4
Ethnicity			
	Han Chinese	743	95.3
	The minorities^a^	37	4.7
Age(year)			
	60-	395	49.2
	70-	320	39.9
	80-	88	11.0
Marital status			
	Married	558	69.8
	Widowed/divorced	242	30.2
Educational level			
	Illiteracy	176	21.9
	Elementary school	207	25.8
	Junior middle school	207	25.8
	Senior middle school	148	18.4
	College or above	65	8.1
Employment status			
	Employed	16	2.0
	Retired	661	82.7
	Others^b^	122	15.3
Health insurance status			
	No health insurance	115	14.3
	At least one	688	85.7
Living pattern			
	Living alone	112	14.0
	Living with others	689	86.0
Personal monthly income(RMB)			
	0-1000	219	28.8
	1001-2000	419	55.1
	2001-3000	70	9.2
	>3000	52	6.8
Main source of income			
	Themselves/spouse	694	87.6
	Children	71	9.0
	Others^c^	27	3.4
Person who provide care			
	Nobody	168	21.5
	Spouse	358	45.8
	Children	221	28.3
	Others^d^	35	4.5

^a^ minority in our survey includes the Hui nationality and Manchu;

^b^ others include unemployed, self-employed and farmers;

^c^ the others include grandchildren, relatives, friends, welfare and mixed source;

^d^ the others include grandchildren, relatives, friends and nanny.

^*^ missing exists, if the sum of n is less than 803

### Participants’ Chronic Diseases Knowledge and Related Factors

The correct rates of chronic diseases knowledge varied from 29.5% to 90.2% (Table 2). Several healthy lifestyles were most well known: maintain a balanced diet (90.2%), quit smoking and drink less (89.2%), keep broad-minded (86.7%), and moderate physical activity (86.3%). The least known knowledge were 2 complications of hypertension: nephropathy (29.5%) and retinopathy (37.2%). The awareness rates of threshold value of BP and normal fasting plasma glucose and warning signs of cancer were also low (from 40.0% to 56.8%).

**Table 2:** Awareness rates of chronic diseases knowledge among the elderly in Jinan.

Questions/knowledge		Correct response	
		n	%
Healthy lifestyle			
	Quit smoking and drink less	716	89.2
	Maintain a balanced diet	724	90.2
	Keep open-minded	696	86.7
	Keep fit	490	61
	Moderate physical activity	693	86.3
Warning signs of cancer			
	Unfamiliar lump	456	56.8
	Abnormal bleeding	383	47.7
	Unexplained weight loss	369	46
	Fatigue whole body	326	40
Threshold value of systolic BP		338	42.1
Threshold value of diastolic BP		366	45.6
Risk factors of hypertension			
	Smoking	429	53.4
	Genetic factors	529	65.9
	Eat more salt	594	74
	Obesity	521	64.9
	Drinking a lot	455	56.7
	Mental strains	411	51.2
	Lack of exercise	322	40.1
Complication of hypertension			
	Stroke	535	66.6
	Nephropathy	237	29.5
	Retinopathy	299	37.2
Control measures of hypertension			
	Take medicine according to the instruction	644	80.2
	Reduce the salt intake	592	73.7
	Moderate exercise	476	59.3
	Keep fit	375	46.7
	Alleviate deleterious psychosocial factors	468	58.3
	Drinking less	399	49.7
	Quit smoking	364	45.3
The threshold value of normal fasting plasma glucose		434	54
Eating and drinking more, polyuria accompanied by weight loss are the typical symptoms of diabetes		600	74.7
Diabetics should control diet and increase physical exercise		563	70.1
Complications of diabetes include hypertension, coronary heart diseases, eye disease and kidney disease		561	69.9
Obese people are susceptible to diabetes		500	62.3
Children are susceptible to diabetes if their parents are diabetics		490	61
People eating too much fish and meat are susceptible to diabetes		498	62

Bivariate analysis showed that higher total knowledge was related to Han Chinese (*P*=0.06), employment status (*P*<0.001), health insurance status (*P*<0.001), main source of income (*P*=0.003), people who provide care (*P*<0.001), family history of chronic diseases knowledge (*P*<0.001), frequency of participating in community activities (*P*<0.001), frequency of communication with neighbors (*P*=0.009), actions taken when ill (*P*=0.031), different medical facilities which the participants usually went to (*P*<0.001). Those of younger age, with higher educational level, higher personal income, and higher frequency of gathering with friends or relatives tended to have more health knowledge (*P*<0.001). People participating in at least one society had more chronic diseases knowledge than those participating in none (*P*<0.001). There were no significant differences of total knowledge for gender, marital status, living pattern and suffering from chronic diseases (*P*>0.05). Results are presented in Table 3 and Table 4.

**Table 3:** Comparison of total knowledge among the elderly in Jinan with different characteristics.

Factors		Mean total knowledge score	*P* (K–W test/M-W U-test)
Gender			
	Male	20.93	0.624
	Female	20.98	
Ethnicity			
	Han Chinese	21.31	0.006
	The minority	17.57	
Age(year)			
	60-	22.36	<0.001
	70-	20.18	
	80-	17.52	
Marital status			
	Married	21.44	0.066
	Widowed/divorced	19.86	
Educational level			
	Illiteracy	17.36	<0.001
	Elementary school	20.22	
	Junior middle school	22.49	
	Senior middle school	22.55	
	College or above	24.55	
Employment status			
	Employed	21.75	<0.001
	Retired	21.81	
	Others	16.57	
Health insurance status			
	No health insurance	16.43	<0.001
	At least one	21.72	
Living pattern			
	Living alone	19.49	0.128
	Living with others	21.19	
Personal monthly income(CNY^*^)			
	0-1000	18.68	<0.001
	1001-2000	21.79	
	2001-3000	23.04	
	>3000	23.71	
Main source of income			
	themselves/spouse	21.44	0.003
	Children	17.75	
	Others	18.70	
Persons who provide care			
	Nobody	20.10	<0.001
	Spouse	22.46	
	Children	19.83	
	Others	18.51	
Family history of chronic diseases			
	Yes	23.31	<0.001
	No	20.46	
Suffering from chronic diseases (self-reported)			
	Yes	21.19	0.813
	No	20.91	

*Note*. ^*^ CNY: Chinese Yuan; 6.2CNY = 1USD

**Table 4:** Comparison of total knowledge among the elderly in Jinan with different behaviors.

Factors		Mean total knowledge score	*P* (K–W test/M-W U-test)
Participating in societies			
	Participating in at least one society	22.91	<0.001
	None	20.43	
Frequency of participating in community activities			
	Never	20.84	<0.001
	1-3 times/year	20.7	
	>4 times/year	25.6	
Frequency of gathering with friends/relatives			
	Scarcely	18.11	<0.001
	Several times a year	20.97	
	Monthly	23.35	
	At least Weekly	23.46	
Frequency of communicating with neighbors			
	Scarcely	19.58	0.009
	Several times a year	15.83	
	Monthly	24.41	
	Weekly	23.1	
	Everyday	21.15	
Actions taken when ill			
	See a doctor immediately	20.98	0.031
	Go to pharmacies to buy medicine	21.37	
	Wait for a while	18.46	
Medical facilities which they usually go to			
	Provincial hospitals/hospitals affiliated with medical universities	22.65	<0.001
	Municipal hospitals	20.74	
	District hospitals	17.31	
	Community health service center/station	19.34	
	Others^*^	17.65	

*Note*. ^*^ the others include private clinics, pharmacy, clinics affiliated with company or working place and no medical institutions to go.

A multivariate logistic regression was conducted to find the related factors associated with higher knowledge (Table 5). Participants with the following characteristics or behaviors were more likely to have higher chronic diseases knowledge: younger age, female, Han Chinese, higher level of education, having health insurance, participating in societies, having family history of chronic diseases, frequently gathering with friends/relatives, usually going to provincial hospitals/hospitals affiliated with medical universities, usually going to municipal hospitals and usually going to community health center/station.

**Table 5:** Multivariate logistic regression analysis on related factors of higher knowledge among the elderly in Jinan.

Factors		B	*P*	OR	95.0% CI
Age (every 10-year reduction)		0.440	0.011	1.55	1.11-2.18
Gender					
	Male			1	
	Female	0.510	0.008	1.67	1.15-2.42
Ethnicity					
	The minorities			1	
	Han Chinese	0.822	0.047	2.28	1.01-5.11
Educational level (every 1-level increment)		0.198	0.014	1.22	1.04-1.43
Health insurance status					
	No health insurance			1	
	At least one	0.591	0.017	1.81	1.11-2.94
Participating in societies					
	None			1	
	Participating in at least one	0.413	0.039	1.51	1.02-2.24
Family history of chronic diseases					
	No			1	
	Yes	0.555	0.010	1.74	1.14-2.65
Frequency of gathering with friends/relatives (every 1-level increment)		0.408	<0.001	1.50	1.27-1.78
Medical facilities which they usually go to					
	The others			1	
	Provincial hospitals/hospitals affiliated with medical universities	1.173	0.001	3.23	1.66-6.28
	Municipal hospitals	0.840	0.044	2.32	1.02-5.24
	District hospitals	-0.243	0.619	0.78	0.30-2.05
	Community health center/station	0.779	0.028	2.18	1.09-4.37
Constant		-4.639	<0.001		

### Major Source of Health Knowledge

Participants mainly acquired their health knowledge from television (77.8%), newspapers or magazines (38.0%) and broadcast (35.2%). Only 31.8% participants acquired health knowledge from doctors. When being asked about the most expected way to get health knowledge, participants raised the following three ways most frequently: television (35.8%), doctors (27.0%), and newspapers or magazines (9.4%).

### The Attitude and Belief to Change Bad Living Habits

Of total participants, 75.4% had confidence to change bad living habits if they were given some help, 19.8% were not sure if they could change, and only 4.8% of participants did not have any confidence to change. Regarding factors that could help to change bad habits, the most frequent answers were doctor’s advice (72.8%), followed by poor health status (54.7%), and then regular physical examination (24.4%).

## Discussion

The vast majority of participants knew healthy lifestyle (from 61.0% to 90.2%). However, lots of old people didn’t know the relationship between healthy lifestyle and chronic diseases. About half of participants didn’t know smoking, drinking a lot, mental strains and lack of exercise were risk factors of hypertension, and about 40% didn’t know high-calorie diet or obesity were risk factors of DM. Most old people believed medicine and reduced salt intake were control measures of hypertension, while only about half of them considered healthy lifestyle as effective. These findings agreed with other studies in China. For example, investigations in Guangzhou and Beijing reported that more than 90% old people knew eating too much salt could elevate high BP, while only about 62% knew lack of physical exercise was one risk factor of hypertension [28,29]. Another study in Yinchuan city, Ningxia Hui Autonomous Region reported that about two-thirds of old people knew diet, smoking, drinking and obesity were related with hypertension and DM [33]. Since healthy lifestyle played an important role in preventing and controlling hypertension, DM and cancer [12], it’s necessary for more people to know this information.

Among all cancers, the prevalence of stomach cancer (18.91%) was highest in Shandong province, followed by lung cancer (16.76), breast cancer (11.27%), esophagus cancer (11.06%), liver cancer (9.34%), colorectal cancer (8.74%), carcinoma of the corpus uteri (4.90%), leukemia (2.85%), ovarian cancer (2.10%), cervical cancer (1.83%) [35]. The cancer prevalence and mortality of old people were much higher than young people [9,35], and it would be much better if the cancers could be detected and treated early. People must be alert to warning signs such as unfamiliar lump, fatigue, abnormal bleeding and unexplained weight loss. A survey in Guangxi Zhuang Autonomous Region showed that 56.5% people knew unfamiliar lump was one of warning signs of cancer, while less than a quarter knew other warning signs like the abnormal bleeding and unexplained weight loss [32]. In our study, the awareness rates of warning signs of cancer were also low (from 40.0% to 56.8%). It was reported that main reasons for subjects unwilling to participate in liver cancer screening were “not knowing the benefits of the screening” (39.7%) and “no symptoms” (28.1%) [32]. In another study, about two-thirds subjects raised the two reasons above for not participating in esophageal cancer screening [36]. Thus together with other studies, our study showed that people including the elderly did not recognize the benefits of cancer screening and knew little about warning signs of cancer. Therefore, education programs on warning signs of cancer as well as promotion of cancer screening need to be strengthened in order to prevent and control cancers in China.

Patients’ knowledge of target BP was independent predictor of successful BP control [15,16]. Since hypertension usually does not cause symptoms, it is necessary for old people especially patients to know the normal BP and check their BP regularly. On the other hand, if one’s high BP is not well controlled, stroke, nephropathy and retinopathy might occur. Thus knowing the complications of hypertension could lead to better medication adherence and better control of hypertension. In our study, however, only about 45% old people knew the threshold values of BP, which was lower than the reports in Guangzhou and Beijing (about 50%) [28,29], and higher than other studies (varied from 14% to 17%) [37–39]. Regarding complications of hypertension, 66.6% participants in our study knew stroke while only about one third knew nephropathy and retinopathy. It was reported that about half of old people in Guangzhou and Beijing knew the complications of hypertension [28,29]. Another study in Guangzhou reported that 70.1% old people knew hypertension could lead to stroke, and only 37.3% knew another complication – kidney damage [30].

In our study, age, gender and educational level were independent factors related to higher knowledge. This was consistent with other studies on diabetes knowledge [40–42]. The group with older age or low educational level had limit abilities to get health knowledge, thus they must be paid more attention in health education. Female was a positive predictor of higher knowledge, and this conflicted with the results of bivariate analysis. After further analysis, we found that: there were no significant difference of ethnicity, age, health insurance status, the frequency of gathering with friends/relatives, family history of chronic diseases and the medical facilities which they usually went to between male and female participants (*P*>0.05); male participants had higher educational level than female participants (*P*<0.05); more male participants participated in societies than female participants (34.2% versus 20.3%, *P*<0.05). This suggested that male participants would have more chronic diseases knowledge if female was not a positive predictor of higher knowledge. Because of the confounding factors above, the total knowledge between male and female participants had no significant difference.

Bivariate analysis showed that old people who participated in any society, had higher frequency of participating in community activities or had higher frequency of gathering with friends/relatives/neighbors had higher level of chronic diseases knowledge. However, in multivariate logistic regression, only participating societies and frequency of gathering with friends/relatives were predictors for higher knowledge. It seemed that old people got more chronic diseases knowledge from societies and friends/relatives rather than neighbors. Thus, it is useful to encourage the elderly to participating in societies and educate their friends/relatives who are younger or have a higher level of education, so that they can educate the elderly and improve their chronic diseases knowledge.

A comparison of health education conducted by different levels of health service institutions showed that the effect of education held by the third graded hospitals (indicating higher level of hospital) was better [43]. Our study showed that participants who usually went to provincial hospitals/hospitals affiliated with medical universities had highest knowledge, followed by municipal hospitals. This suggested that lower level medical facilities need to improve their skills of health education. In addition, usually going to community health center/station was one of predictors of higher knowledge (OR=2.18, 95% CI: 1.09-4.37), which might be attributed to health education carried out in community health center/station.

Having health insurance was also one predictor of higher knowledge (OR=1.81, 95% CI: 1.11-2.94). Some patients didn’t go to hospital because of high medical costs [44]. It’s obvious that health insurance helped to reduce economic burden of diseases greatly. So those having no health insurance probably got less contact with doctors and received less health-related information from doctors. According to the fourth national health services survey [45], there were still 28.1% urban residents who had not any health insurance. In order to improve the health of the elderly, health insurance coverage must be further expanded.

Bivariate analysis showed that old people with family history of chronic diseases had more knowledge than those with none (*P*<0.001), and old people suffering from chronic diseases did not had significantly higher knowledge (*P*>0.05). In multivariate analysis, family history of chronic disease was one of predictors of higher knowledge. A study in Guangzhou city, Guangdong province reported that history of suffering from DM, family history of DM and family history of hypertension were related with high DM knowledge (OR=2.752, 1.897 and 1.373 respectively) [42]. Possible reasons for this contradiction include: (1) these factors were affected by some confounding factors which were not analyzed in their study; (2) our study included other knowledge besides DM knowledge; and (3) subjects were different in their study (residents above 25 years old).

Television (35.8%) and doctors (27.0%) were the major expected sources of acquiring health knowledge, which agrees with other studies in China [33,46]. Therefore, various scientific TV programs about health education are in great need to improve the elder’s chronic diseases knowledge. Koo’s study showed that physician recommendation was significant predictors of colorectal cancer screening test uptake [18]. Other studies also showed that visits to dieticians was independently associated with greater diabetes knowledge [40], and not having visited a physician within the preceding 12 months was independent predictor of a lack of awareness of hypertension [47]. In our study, 72.8% participants thought doctor’s advice could help them to change bad habits. Doctors’ advice played an important role in health education and health promotion. Thus, doctors, especially general practitioner in community health centers, ought to strengthen health education and guidance to the elderly.

This study had some limitations. First, this was a cross-sectional study and there was possibility of temporal ambiguity. Some factors related to high level of chronic diseases knowledge might be results rather than causes of knowledge. For example, buying health insurance and going to higher level hospitals. Second, there was possible selection bias because some eligible subjects did not consent to participate and some could not participate for various reasons. Third, psychometric properties of the disease knowledge questionnaire were comparatively weak. Fourth, because of the small number in some cells (for example, employment status ‘employed’), the results might be unstable. Lastly, this study used a newly developed instrument and was carried out in Jinan, the provincial capital of Shandong province, where the people had a higher level of living and better medical health conditions than those in small cities, thus the findings had limited generalizability. Further studies are needed to discover status of old people’s chronic knowledge and its related factors.

In conclusion, old people in Jinan had incomplete chronic diseases knowledge and the overall awareness rate was not high. Therefore, it’s urgent to improve the old people’s chronic diseases knowledge, especially the knowledge about complication of hypertension, threshold value of normal BP, warning signs of cancer and the relationship between healthy lifestyle and chronic diseases. Younger age, female, Han Chinese, higher educational level, having health insurance, participating in societies, having family history of chronic diseases, frequently gathering with friends/relatives, usually going to provincial hospitals/hospitals affiliated with medical universities, usually going to municipal hospitals, and usually going to community health center/station were independently associated with higher chronic diseases knowledge. The old people showed positive attitude to change bad living habits. Health education programs should target those who are male, older, minorities, with lower educational level, having no health insurance, having no family history of chronic diseases, participating in no societies, and less frequently gathering with friends/relatives. Besides, lower level medical facilities should improve their skills of health education.
